# Genes and Co-Expression Modules Common to Drought and Bacterial Stress Responses in *Arabidopsis* and Rice

**DOI:** 10.1371/journal.pone.0077261

**Published:** 2013-10-10

**Authors:** Rafi Shaik, Wusirika Ramakrishna

**Affiliations:** Department of Biological Sciences, Michigan Technological University, Houghton, Michigan, United States of America; National Institute of Environmental and Health Sciences, United States of America

## Abstract

Plants are simultaneously exposed to multiple stresses resulting in enormous changes in the molecular landscape within the cell. Identification and characterization of the synergistic and antagonistic components of stress response mechanisms contributing to the cross talk between stresses is of high priority to explore and enhance multiple stress responses. To this end, we performed meta-analysis of drought (abiotic), bacterial (biotic) stress response in rice and *Arabidopsis* by analyzing a total of 386 microarray samples belonging to 20 microarray studies and identified approximately 3100 and 900 DEGs in rice and *Arabidopsis*, respectively. About 38.5% (1214) and 28.7% (272) DEGs were common to drought and bacterial stresses in rice and *Arabidopsis*, respectively. A majority of these common DEGs showed conserved expression status in both stresses. Gene ontology enrichment analysis clearly demarcated the response and regulation of various plant hormones and related biological processes. Fatty acid metabolism and biosynthesis of alkaloids were upregulated and, nitrogen metabolism and photosynthesis was downregulated in both stress conditions. WRKY transcription family genes were highly enriched in all upregulated gene sets while ‘CO-like’ TF family showed inverse relationship of expression between drought and bacterial stresses. Weighted gene co-expression network analysis divided DEG sets into multiple modules that show high co-expression and identified stress specific hub genes with high connectivity. Detection of consensus modules based on DEGs common to drought and bacterial stress revealed 9 and 4 modules in rice and *Arabidopsis*, respectively, with conserved and reversed co-expression patterns.

## Introduction

Crop productivity and survival is tightly linked to its environment which is being altered due to climate change, biodiversity loss and degradation of land and freshwater [1] threatening the food security of the world while the food demand is estimated to increase by 70% in 2050 [2,3,4]. According to latest World Agricultural Supply and Demand Estimates (WASDE) report by United States Department of Agriculture (USDA), about 80% of agricultural land is experiencing drought and over 2,000 U.S. counties had been designated as disaster areas [5]. Reflecting the declining environmental conditions, more often than not plants today are exposed simultaneously to multiple stresses resulting in enormous changes in the molecular landscape within the cell. Comprehensive understanding of the regulatory networks that modulate the dynamic adaptive changes in a plant responding to stress is critical to meet future energy needs. Rice and *Arabidopsis* are both model plant organisms representing monocots and dicots respectively. Both the plants have extensive biological knowledgebase and resources including complete genome sequence and highest number of microarray studies in the plant kingdom. Thus, analysis of stress responsive genes within and between rice and *Arabidopsis* for different kinds of stresses would reveal a number of pivotal attributes spanning across the major plant division, angiosperms.

 Advancements in high throughput technologies have resulted in deluge of various kinds of -omic data addressing different aspects of temporal and spatial response in variety of stresses in plants. Microarray technology revolutionized the identification of global transcriptomic changes and today multiple transcriptomic studies exist for the same or related stress conditions. Thus meta-analysis of related microarray studies is increasingly becoming popular to enhance the sensitivity of the hypothesis addressed and validate conclusions [6]. So far, very few meta-analysis studies are available in plant systems [7,8,9,10,11]. Meta-analysis of microarray data from *Arabidopsis* infected with eight different viruses revealed hub genes that are highly connected, organized in modules and are central to plant defense response [12]. It is reported that in plants responding to multiple stresses, there exists extensive cross-talk between different stress responses via hormonal signaling pathways [13]. Thus, it is imperative to compare and analyze different kinds of stress responses to find the genes, proteins and metabolites that are common and specific to different kinds of abiotic and biotic stress conditions. Meta-analysis of microarray studies involving samples from a wide range of tissues, developmental stages and different levels of stresses but specific to one stress condition would unravel the universal principles and features related to the stress response. Comparative analysis of such universal molecular profiles from different stresses would allow the identification of unique and shared features. Further, comparison of the stress responsive profiles across diverse plant species would reveal the conserved stress specific mechanisms and uncover orthologous genes that are most critical to the stress response. 

Recently, there has been an upsurge in the number of studies reporting global co-expression networks of plants based on genome wide transcriptome data [14,15,16]. A number of tools namely ATTED-II [17], CressExpress [18], RiceArrayNet [19], OryzaExpress [20] and RiceFREND [21] based on co-expression networks are available that can be explored to identify novel genes, predict gene functions and characterize gene regulatory networks. A network based analysis in rice identified drought responsive gene modules and found a module with 134 genes specifically associated with both drought tolerant and drought resistant rice varieties [22]. Weighted Gene Co-expression Network Analysis (WGCNA) is one of the latest and popular methodologies to decipher correlation patterns across microarray samples [23]. Implemented in R as a package, WGCNA provides a vast array of functions to detect, analyze and export individual and consensus modules from diverse but related microarray studies. WGCNA has been utilized to detect coexpression modules in *Arabidopsis*, rice, maize, soybean and poplar [14,24,25] and also across species [26].

In this study, we performed large scale comparative transcriptomic analysis via meta-analysis of microarray data on drought and bacterial stress in rice and *Arabidopsis*. To elucidate the cross talk between different stress conditions, knowledge of the expression status of genes involved in stress response is critical. Our analysis revealed the genes that are unique to each stress and those that are shared with other stress conditions. Further, within common genes, we also found genes that were up or downregulated in both stresses and also genes which showed reversed expression status. Extensive analysis of various gene sets based on Gene Ontologies (GO), KEGG Orthologies (KO) and metabolic pathway analysis unraveled the underlying biological mechanisms related to different stresses. We then performed co-expression network analysis which divided the stress responsive genes into tightly co-expressed modules revealing organization of stress transcriptome.

## Methods

### Selection of stress related microarray studies

Gene Expression Omnibus (GEO) is the central repository for microarray and other forms of high-throughput data [27]. Experiments conducted on the Affymetrix platforms, Rice Genome Array (GPL2025) and *Arabidopsis* ATH1 Genome Array (GPL198) were chosen for this study as they provide extensive gene coverage and are widely used. GEO currently holds 1920 and 9106 samples and 114 and 709 series records (group of related samples) belonging to GPL2025 and GPL198 platforms, respectively. In total, we analyzed 305 and 220 samples of rice and *Arabidopsis*, respectively, belonging to 28 series records. Different series involving samples from various tissues, developmental stages and stress levels were included to identify differentially expressed genes in a wide range of conditions with higher sensitivity and uncover an overarching drought and bacterial stress response profile. The number of selected series, sample records and number of controls and treatments for each stress condition is given in Table 1. Complete list of selected series and sample records including their GEO IDs and brief description is given in Table S1 in File S1. 

**Table 1:** Number of microarray studies for each stress condition.

**Stress condition**	**GEO Series**	**GEO Samples**	**No. of controls**	**No. of treatments**
**RD**	5	78	35	43
**RB**	7	227	61	166
**AD**	7	114	61	53
**AB**	5	106	40	66
**Total**	24	525	197	328

RD: Rice Drought; RB: Rice Bacteria; AD: Arabidopsis Drought; Arabidopsis Bacteria

### Identification of differentially expressed genes

The raw intensity CEL files of the selected samples were downloaded from GEO and intensity values were extracted from the CEL files using the bioconductor package Affy in R [28], quality checked using the package, ArrayQualityMetrics [29] and the samples failing two or more of its quality tests were removed. The samples of each stress were normalized together using Robust Multichip Average (RMA) method [30]. The probes were then matched to their loci based on annotation provided by array element mapping facility at TAIR portal for *Arabidopsis* (http://www.Arabidopsis.org/portals/expression/microarray/microarrayElementsV2.jsp) and at ricechip.org (http://www.ricechip.org) for rice. Probes with no match or ambiguously matching multiple loci were discarded. The retained probes and their normalized intensity values were then loaded into oneChannelGUI environment to perform non-specific filtering of probes with relatively small signal distribution using Inter Quartile Range (IQR) filter at most stringent setting (0.5) and probes with very low intensity values (probes below threshold log_2_(50)=5.64 in ≥90% of arrays). An example of resultant distribution of retained probes after filtering is shown in Figure S1. 

Differentially expressed genes (DEGs) were identified using Rank Product method [31]. Rank Product is a non-parametric method returning up and down regulated genes, their fold change (FC), p-values and percentage of false predictions (PFP). It was shown to perform better than other methods including significance analysis of microarrays (SAM), Fisher’s Inverse χ^2^ test and t-based hierarchical modeling [32] and is widely used for meta-analysis studies combining data sets from different origins of the sample pool to increase the power of identification [6]. We used the function RPadvance of the bioconductor package RankProd [33] which is specifically designed for meta-analysis. The number of permutation tests was set to 250. The function topGene with a PFP cut-off value of ≤0.01 was used to output differentially expressed genes. Among multiple probes matching the same locus, the probe ID with highest fold change was retained. 

The orthologs between rice and *Arabidopsis* were obtained by parsing the gene families reported in GreenPhylDB [34] which were identified based on analysis of complete proteomes of 16 plant species, cross referencing a number of resources (UniProtKB, Pubmed, InterPro, MEME motifs, KEGG pathways).  

### Functional enrichment analysis

Gene ontology analysis was carried out using the Singular Enrichment Analysis (SEA) tool offered by agriGO [35] at default settings of Fisher t-test (p<0.05), False Discovery Rate (FDR) correction by Hochberg method and five minimum number of mapping entries against species specific pre-computed background reference. KEGG orthology (KO) terms associated with a gene correspond to KEGG pathway nodes and BRITE hierarchy nodes [36]. To identify enzymes and proteins encoded by differently expressed genes and their associated metabolic and signaling pathways in each stress condition, we performed enrichment analysis of KO terms and determined the significance based on hypergeometric distribution p-values with <0.05 cut off value. Further analysis of biological pathways was carried out using the tool Database for Annotation, Visualization and Integrated Discovery (DAVID) v6.7 [37]. Information on transcription factors (TFs) genes in rice and *Arabidopsis* was obtained from the database PlnTFDB [38] and analyzed for enrichment of TF families in stress responsive genes. 

### Co-expression network analysis

To identify co-expression modules within SRGs, we extracted the normalized, log transformed gene expression values of each stress condition from the microarray experiments used in meta-analysis and performed Weighted Gene Co-expression Network Analysis (WGCNA) [23]. Briefly, WGCNA procedure calculates Pearson’s correlation matrix for all genes, transforms the correlation matrix by raising all values to a power ß (soft thresholding as biological networks are small world and scale free [39]), calculates a topological overlap matrix (TOM) from the transformed correlation matrix, converts the topological overlap matrix into a dissimilarity matrix, creates a hierarchical cluster tree based on the dissimilarity matrix, and identifies gene co-expression modules from the hierarchical cluster tree using a dynamic tree cut procedure. The blockwiseModules function of WGCNA package in R was used to generate the modules with powers 8, 6, 14 and 5 for RD, RB, AD and AB, respectively, which best approximate a scale-free topology (model fit >0.8) of the resultant network (Figure S2). For this analysis, module size was 20-30, deep split was set at level 4 and tree merge cut height was 0.15-0.25. Heatmaps were constructed to depict the eigengenes from each identified module. Eigengenes represent a centroid measure of the expression levels of all genes in a cluster. The SRGs common to drought and bacterial stress were analyzed to find consensus modules showing co-expression patterns across stresses using the function blockwiseConsensusModules with the following settings: powers 7 and 10, minimum module size 30 and 15 for rice and *Arabidopsis*, respectively, with the merge cut height set at 0.15. 

## Results and Discussion

### Highly conserved expression status of genes common to drought and bacterial stresses

**Figure 1:**
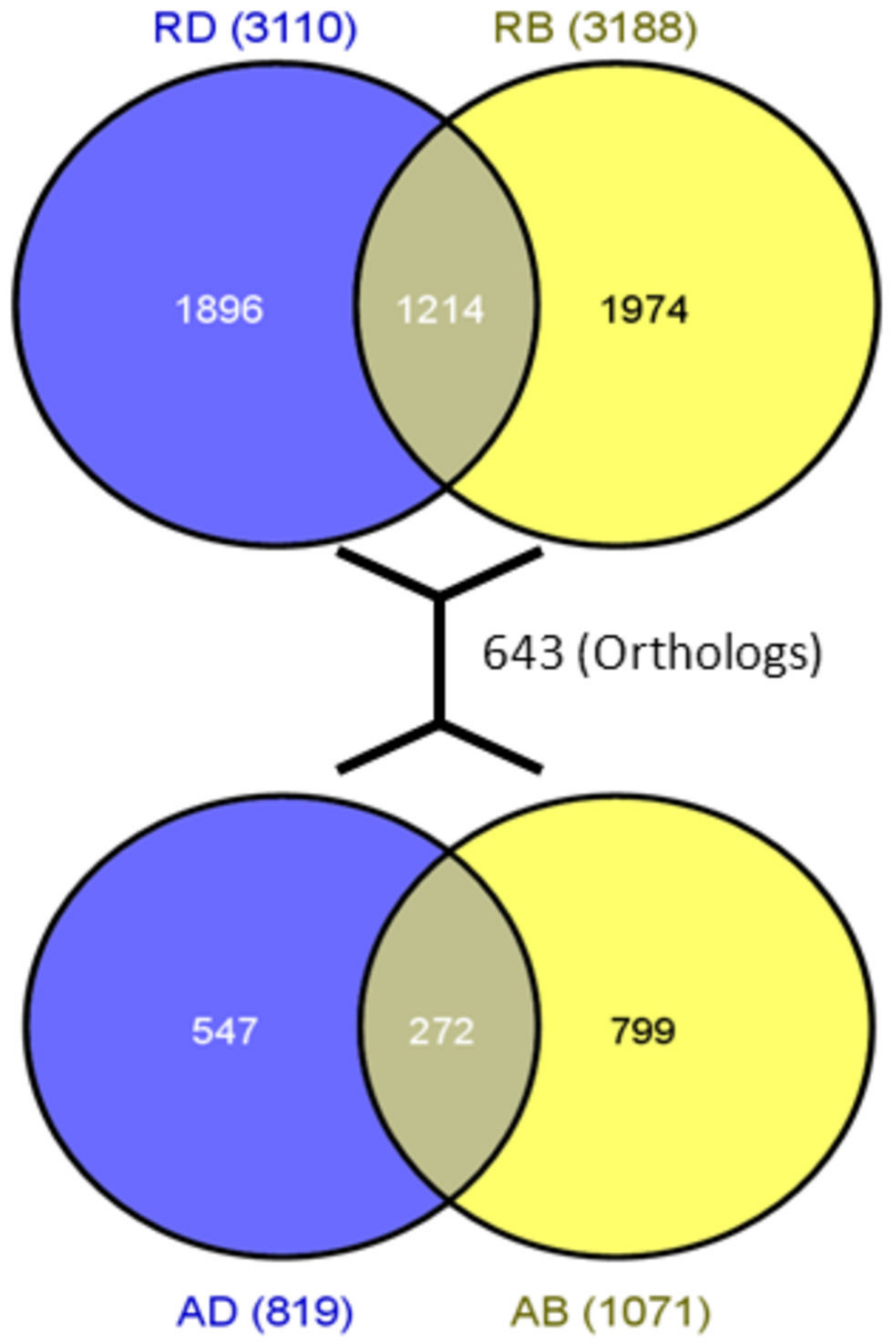
Number of unique and common differentially expressed genes (DEGs) found in rice and *Arabidopsis*. The number of orthologous genes found between rice and *Arabidopsis* DEGs are also shown. RD: Rice Drought, RB: Rice Bacteria, AD: *Arabidopsis* Drought, AB: *Arabidopsis* Bacteria.

We identified a total of 5084 and 1618 DEGs referred herein as stress responsive genes (SRGs) in rice and *Arabidopsis*, respectively, combining the DEGs in drought and bacterial stresses together that were below PFP ≤0.01 (Figure 1). Greater than 60% of genes were unique to individual stresses in all cases and AB (*Arabidopsis* Bacteria) had highest percent (~75%) of unique SRGs (799 genes). The number of up and downregulated SRGs are shown in Figure S3A and complete list of genes along with their fold change values is given in Table S2 in File S1. Among the 1214 SRGs common to the stresses studied in rice, majority of the genes were expressed in same direction (72% or 874) with 565 up and 309 downregulated in both drought and bacterial stresses. Similarly, higher number of SRGs (170 out of 272 or 62.5%) common to both stresses studied in *Arabidopsis* was expressed in same direction with 93 and 77 genes up and downregulated, respectively. This finding elucidates that these set of genes and their associated biological processes are altered similarly as part of stress response in a wide range of tissues, developmental stages, stress levels and ecotypes (Table 1A). Among the genes with non-conserved expression pattern, the proportion of genes showing downregulation in drought and upregulation in bacterial stress (255 or 21% of 1214) was higher in rice while upregulation in drought and downregulation in bacterial stress (66 or 24% of 272) was higher in *Arabidopsis* (Figure S3D). Further comparison of PFP values of common SRGs revealed that about 30% (371 out of 1214) and 15% (33 out of 272) of rice and *Arabidopsis* genes, respectively, showed very high significance in both the stresses (PFP <0.0001). Especially among common SRGs of rice, ~40% (121/309) of genes that showed conserved upregulation were with PFP <0.0001 in both the stresses.

 To further validate the reliability of the identified genes, we compared the DEGs against lists of genes that showed stable expression in multiple studies. None of the 1618 *Arabidopsis* DEGs were part of 39 genes proposed as a superior set of reference genes for the normalization of gene expression by Czechowski et. al [40]. Two of the 5084 rice DEGs, LOC_Os08g23180 coding for arabinogalactan protein found only in RD DEGs and LOC_Os02g38920 coding for GAPDH found only in RB DEGs are part of 26 genes reported by Narsai et. al. showing stable expression [41], which probably is due to the limited number of tissues, developmental stages and stress treatments used in their analysis. 

The average fold change observed for SRGs was about 1.52, 0.93, 1.28 and 0.99 for RD (Rice Drought), RB (Rice Bacteria), AD (*Arabidopsis* Drought) and AB (*Arabidopsis* Bacteria) stresses respectively. The number of SRGs with fold change (FC) value ≥1.5 was higher in drought stress (51% and 26% in RD and AD respectively) and lower in bacterial stress (4% and 3% in RB and AB respectively), majority of which were part of downregulated genes. Especially three genes showed >11 fold downregulation in RD, with LOC_Os05g47540 annotated as ‘CPuORF26 - conserved peptide uORF-containing transcript, expressed’ under expressed 20.86 folds. Upstream open reading frames (uORFs) are small open reading frames found in the 5' UTR of mature mRNA which regulate translation of major ORFs (mORFs) that code for transcription factors, signal transduction factors and developmental signal proteins [42]. Multiple studies have reported the involvement of uORFs in translation repression of target genes in response to stress conditions [43]. We found this gene to be downregulated also in RB (FC 1.55). LOC_Os10g36500 annotated as ‘invertase/pectin methylesterase inhibitor family protein’ is the second top DEG which was downregulated in both stress conditions (FC 11.34 and 1.20 in RD and RB, respectively). Pectin methylesterase inhibitors (PMEI) are invertase inhibitor-related defense proteins that play key roles in developmental transitions, wounding, senescence and abiotic stresses [44]. Another gene that was highly downregulated in RD (FC 11.08) but upregulated in RB is LOC_Os04g39320 annotated as ‘expressed protein’. In all the four stresses about 15-20% of SRGs were annotated as just ‘expressed protein’ or ‘protein_coding’ or ‘unknown protein’ (500, 556, 220, 157 DEGs in RD, RB, AD and AB, respectively) suggesting there are still hundreds of stress responsive genes with little or no functional information. We also found ~1% SRGs (27 and 34 genes in RD and RB, respectively) were annotated as retrotransposon related genes in rice. In *Arabidopsis*, 21 genes showed >4 fold downregulation under drought stress with AT1G22690 annotated as ‘gibberellin-responsive protein’ and AT5G03350, a legume lectin family protein showing 8.8 and 7.9 FC, respectively. 

We found 642 orthologous genes between rice and *Arabidopsis* that are involved in stress response (Table S3 in File S1). There were 255 orthologous genes differentially expressed in drought out of which 167 or 65% had their expression status conserved (73 and 94 were up and downregulated, respectively, in both rice and *Arabidopsis* genomes). Similarly, there were 280 orthologous genes differentially expressed in bacterial stress, out of which 211 or 75% had their expression status conserved. Majority of these were upregulated in both the genomes (134 or 63% SRGs). We also analyzed orthologs between AD and RB, and found 72 SRGs with conserved upregulation. On the other hand, there were 102 SRGs with conserved downregulation between AB and RD (Figure S4). There were 9 up and 8 downregulated orthologous genes found in all four stresses. One of these genes is a MYB TF that was highly downregulated, especially in drought (AT2G21650 (FC 3.5) and LOC_Os01G44390 (FC 5.5)). ARR6 and 7 (two-component response regulators) and their orthologous gene OsRR10 involved in cytokinin response system [45,46] were also downregulated in all stresses. The upregulated genes in all four stress conditions include a NAC TF (AT1G69490 and LOC_Os03G21060), HAI-1 or highly ABA-induced PP2C gene 1 (AT5G59220 and LOC_Os05G38290) and a heavy metal-associated domain containing protein (AT5G52760 and LOC_Os10G38870). Expression of HAI-1 gene was shown to be induced by wound in *Arabidopsis* [47].

### Comparison of significant gene ontology terms of SRG sets distinguished the roles of different hormones and related processes

**Figure 2:**
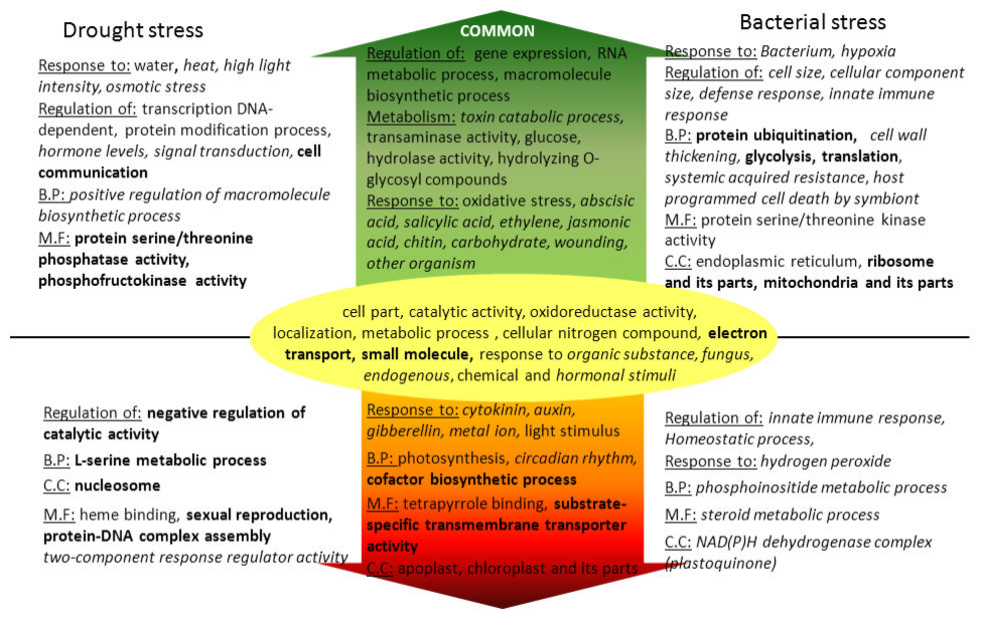
Summary of significant GO terms found in different stresses. Terms in green arrow indicate those that are commonly upregulated in drought and bacterial stress, and the terms besides the green arrow indicate those that are specifically upregulated in one stress. Similarly the terms in red arrow and those besides indicate the terms that are downregulated in both stresses and specific to one stress respectively. Terms in yellow oval were found both in up and downregulated gene sets. The terms in bold and those in italics are highly significantly found in rice and *Arabidopsis* respectively. B.P: Biological Process, M.F: Molecular Function, C.C: Cellular Component.

We found 623 unique GO terms enriched by SRGs in one or more stress conditions (Table S4 in File S1). We analyzed gene sets that are up or downregulated separately for each stress as shown in Figure S3B. Although the number of SRGs in *Arabidopsis* was only 1/3^rd^ compared to those found in rice, total number of significant GO terms in *Arabidopsis* is close to rice reflecting the lack of annotation for a number of rice genes. Four way Venn diagram analysis revealed the number of GO terms common and exclusive to same stress (28 terms between RBU and ABU vs. 4 terms between RBU and ADU) and same species (68 between ADU and ABU vs. 10 between ADU and RDU) were higher than vice versa (Figure S5). The top most significant GO term found in upregulated gene sets were response to water (FDR 5E-11), ribosome (2.9E-37), response to organic substance (3.4E-31) and response to biotic stimulus (2.4E-30) in RDU, RBU, ADU and ABU, respectively and in downregulated sets were catalytic activity (1.7E-24), photosynthesis (1.7E-16), thylakoid (2.6E-18) and response to chemical stimulus (1.3E-15) in RDD, RBD, ADD and ABD, respectively. The terms, ‘polysaccharide catabolic process’, ‘hydrolase activity, hydrolyzing O-glycosyl compounds’, ‘aromatic amino acid family metabolic process’, ‘regulation of gene expression’, ‘transcription factor activity’ were significantly enriched in upregulated gene sets, while ‘photosynthesis’, ‘circadian rhythm’, ‘cofactor biosynthetic process’, ‘substrate-specific transmembrane transporter activity’ were significantly enriched in downregulated gene sets (Figure 2). 

Terms related to hormones and their related functions showed clear distinction between the processes that are up or downregulated in a stress response especially in *Arabidopsis*. While terms related to the hormones auxins, cytokinins and gibberellins were downregulated, abscisic acid, salicylic acid, ethylene and jasmonic acid was upregulated both in drought and bacterial stresses. Abscisic acid (ABA) is known to play a central role in abiotic stress response by inducing stomatal closure resulting in reduction of transpiration [48], regulating root growth, ion channels and gene expression [49]. Further, it was found that ABA can have both positive and negative effect on biotic stress signaling [50]. For example, ABA-induced stomatal closure prevented invasion of microbes through open stomata. Thus, recent findings increasingly suggest ABA as a key player in fine-tuning of cross talk between abiotic and biotic stress responses and therefore ABA production can be the crucial factor determining how well a plant responds to multiple stresses [51]. While the terms ‘response to ethylene stimulus’ and ‘response to salicylic acid stimulus’ were found both in ADU and ABU, the terms ‘ethylene mediated signaling pathway’ and ‘salicylic acid mediated signaling pathway’ were significant only in ABU, which is in agreement with their known functional roles in defense against pathogens and senescence [52,53]. Further, the terms ‘host programmed cell death induced by symbiont’ and ‘systemic acquired resistance (SAR)’ mechanisms that are induced by salicylic acid were also found only in ABU. On the other hand, jasmonic acid biosynthetic process was significant only in ADU although jasmonic acid mediated signaling pathway was significant both in ADU and ABU. Jasmonic acid (JA) plays a key role in defense response especially against necrotrophic pathogens and wounding acting antagonistically to salicylic acid which is majorly involved in resistance to biotrophic pathogens [54]. JA also has a role in the formation of antioxidants that regulate ascorbate and glutathione metabolism [55] explaining our observation of its increased synthesis in drought stress. The downregulation of all of the major plant growth and development promoting hormones such as auxins, cytokinins and gibberellins across diverse stress conditions indicates various processes including cell differentiation, chloroplast biogenesis, flowering and reproduction [56,57], controlled by them are pushed to backseat while processes related to reprogramming of metabolism, gene expression, balancing of homeostasis and modulation of defense and immunity are given higher priority. The above observations are further supported by a number of terms related to photosynthesis and biosynthesis of its components including ‘chloroplast’, ‘photosystem’, ‘photosynthetic membrane’, ‘photosynthesis, light reaction’, ‘photosynthetic electron transport chain’ that were highly enriched in all four downregulated gene sets but none of the upregulated gene sets. 

GO terms related to various metabolic processes including carbohydrates, amino acids, proteins, ribosomes, translation and nucleobases were significantly enriched in RBU. Translation is a highly energy expensive process and its regulation via protein phosphorylation, initiation factor isoforms, RNA sequence element interactions, and small RNAs enable cells to rapidly and reversibly control gene expression in response to environmental changes [58]. Upregulation of a number of translation related GO terms in rice under bacterial stress suggests cellular adjustments at translational level upon bacterial infection. The term ‘response to water’ was highly enriched in RDU (FDR 5E-11) and ADU (FDR 4.6E-19) and the term ‘response to water deprivation’ was highly enriched in ADU (1.5E-18). A number of terms related to regulation of gene expression and metabolic processes including ‘transcription factor activity’, ‘nucleic acid metabolism’, and ‘chitin catabolic process’ were enriched in three or all of the upregulated gene sets. Both positive and negative regulation of response to stimulus was found in ABU. The term ‘negative regulation of defense response’ was also significantly enriched in ABU (FDR 8.5E-05). The SRGs associated with the above GO term, EDS1 (Enhanced disease susceptibility 1) and PAD4 (Phytoalexin deficient 4) directly interact and induce salicylic acid biosynthesis in response to biotrophic pathogens [59]. A mutant of EDS1 was found to be disease resistant [60].

### KEGG pathway analysis of SRGs identified upregulation of fatty acid, aromatic amino acid, glucose metabolism and biosynthesis pathways of secondary metabolites

The enriched KEGG orthology (KO) terms in different SRG sets revealed many similar patterns as that of GO analysis that can be seen by the top KO terms and their associated pathways in Table 2. Enrichment of ‘jasmonate ZIM domain-containing’ proteins (JAZs) and ‘auxin responsive GH3 gene family’ proteins in the upregulated SRGs of *Arabidopsis* substantiate recent findings that these proteins negatively regulate downstream processes of hormonal activity especially those related to plant growth and development [61,62,63]. On the other hand, KO terms, ‘two-component response regulator ARR-A family’ involved in negative regulation of cytokinin signaling via phospho relay [64] and ‘SAUR family proteins’ which are primary auxin-inducible genes involved in auxin transport and organ elongation [65] were enriched in downregulated gene sets of both the stresses. Reactive oxygen species (ROS) have been proposed as a central component of plant adaptation to both biotic and abiotic stresses [66]. Glutathione S-transferase (GST) plays a key role in scavenging ROS and detoxification and is differentially activated by stress-induced plant growth regulators [67]. GST was upregulated in both the stresses and was also part of ADD. 

**Table 2:** Top KO terms and their associated pathways.

**KO ID**	**Definition**	**Pathway**	**Ref No.**	**ADD**	**ADU**	**ABD**	**ABU**
K00430	Peroxidase	Phenylalanine and methane metabolism; Phenylpropanoid biosynthesis	144	11			
K00511	Squalene monooxygenase	Sesquiterpenoid and triterpenoid biosynthesis	6			3	
K00799	Glutathione S-transferase	Glutathione metabolism	46	4	6		7
K09580	Protein disulfide-isomerase A1	-	7				3
K13464	Jasmonate ZIM domain-containing protein	Plant hormone signal transduction; Plant-pathogen interaction	20		7		
K14487	Auxin responsive GH3 gene family	Plant hormone signal transduction	18				4
K14488	SAUR family protein	Plant hormone signal transduction	59	9		5	
K14492	Two-component response regulator ARR-A family	Plant hormone signal transduction	10	4		3	
K14497	Protein phosphatase 2C	Plant hormone signal transduction	9		4		
				**RDD**	**RDU**	**RBD**	**RBU**
K00815	Tyrosine aminotransferase	Phenylalanine, tyrosine and tryptophan biosynthesis	4		3	3	
K02639	Ferredoxin	Photosynthesis	5			4	
K02987	Small subunit ribosomal protein S4e	Ribosome	3				3
K03283	Heat shock 70kDa protein 1/8	Spliceosome; MAPK signaling pathway; Protein processing in ER	6		3		4
K05953	Nicotianamine synthase	-	3	3			
K09874	Aquaporin NIP	-	6	4			
K10999	Cellulose synthase A	Starch and sucrose metabolism	9	6			
K00640	Serine O-acetyltransferase	Cysteine and methionine metabolism	5			3	

A number of terms related to enzymes involved in biosynthetic pathways of amino acids including ‘peroxidase’, ‘tyrosine aminotransferase’ and ‘serine O-acetyltransferase’ were part of downregulated gene sets (Table S5 in File S1). The KO term ‘Cellulose synthase A (CesA)’ was highly enriched in RDD. Several studies reported disruption of genes involved in biosynthesis of cellulose enhanced stress tolerance [68,69,70]. As also revealed by GO analysis, the term ‘small subunit ribosomal protein S4e’ was enriched in RBU and ‘ferredoxin’ involved in photosynthesis was enriched in RBD. Heat shock protein 70 (Hsp70) is one of the most abundant heat shock proteins in eukaryotic cells which bind to hydrophobic patches of partially unfolded proteins preventing protein aggregation [71]. Hsp70 was enriched in both the upregulated gene sets of rice. 

The KEGG pathways found significant by the tool DAVID with p-value <0.05 in SRG sets are shown in Figure S6. The pathway ‘fatty acid metabolism’ was enriched both in RDU and RBU. Plants acclimating to stress modulate membrane fluidity and levels of oleic acid and linolenic acid using lipases facilitating proper functioning of critical integral proteins during stress [72]. α-linolenic acid released under stress from chloroplast membranes is a major parent compound for an array of messenger compounds derived via oxidative modification by ROS [73] including jasmonic acid [74,75]. The pathway ‘α-linolenic acid metabolism’ was highly significant in ADU and RBU. A number of pathways related to biosynthesis of secondary metabolites were enriched in upregulated sets including biosynthesis of alkaloids from shikimates, purines, histidine, terpenoids and polyketides. Phenylpropanoids, derived from a very limited set of core structures of shikimate pathway are modified by oxygenases, ligases, oxidoreductases and transferases to generate an enormous number of secondary metabolites (>200,000) including lignins, suberins and tannins which contribute substantially to the robustness of plants facing stress [76] and are also implicated in providing nutritional and medicinal benefits for animals and humans due to their potent antioxidant activity [77]. Phenylpropanoid biosynthesis was enriched in drought especially in rice but was found both in up and downregulated gene sets suggesting differential regulation of the enzymes resulting in synthesis of different end-products. The biosynthetic pathway of flavonoids from phenylpropanoid derivatives was enriched in ABD. 

Biosynthesis and metabolic pathways of aromatic amino acids, phenylalanine, tyrosine and tryptophan and degradation pathways of lysine, valine, leucine and isoleucine were enriched in upregulated gene sets (ABU, ADU, RBU and RDU). The aromatic amino acids are also synthesized via the shikimate pathway playing crucial roles in plant growth, development, reproduction, defense, and environmental responses [78,79]. Recent reports indicate reduction in starch biosynthesis and accumulation, and increased consumption of storage substances under drought [80,81] resulting in elevated levels of hexose sugars (glucose and fructose) [82]. Our analysis revealed upregulation of starch and sucrose metabolism, glycolysis/gluconeogenesis and pentose phosphate pathway in both drought and bacterial stresses. As observed in GO analysis, a number of pathways related to photosynthesis were enriched in downregulated gene sets including porphyrin and chlorophyll metabolism, carbon fixation in photosynthetic organisms and carotenoid biosynthesis. Similar to the observation of enrichment of GO term ‘nucleobase, nucleoside, nucleotide and nucleic acid biosynthetic process’ in RDD, ‘amino sugar and nucleotide sugar metabolism’ pathway was also enriched in RDD. 

### Differential enrichment ‘CO-like’ TF family members under drought and bacterial stresses

Out of the 82 and 56 known TF families/regulators, 34 (41%) and 38 (67%) were found in one or more gene sets of *Arabidopsis* and rice, respectively (Figure S3C). A comparative list of the number of TFs belonging to each TF family found in different stresses is given in Table S6 in File S1. Among the large TF families, higher numbers of NAC, ERF, AP2-EREBP and C2H2 family members were found in upregulated gene sets while higher numbers of bHLH and MYB_related family members were found in downregulated gene sets. WRKY TFs were the highest in the upregulated set of bacterial stress both in rice and *Arabidopsis*. WRKY TFs are considered to be at the heart of global regulation of plant immunity by modulating its immediate downstream target genes which include MAP kinases and other TFs [83]. ‘CO-like’ TF family members were the highest in RBU (17 TFs) and RDD (11) but low in RBD (1) and RDU (3) indicating an inverse expression relationship between drought and bacterial stress.  *CO* (*CONSTANS*) gene and other members of CO-like TF family play an important role in regulation of flowering and act between the circadian clock and genes controlling meristem identity [84]. A high number of HSF (heat shock transcription factor) family members were found in upregulated gene sets of rice. Seven HD-ZIP (heomodomain leucine zipper motif) members were found in RDU only. Out of 16 Tify family members in *Arabidopsis*, seven were found in ADU. Tify is a novel TF family with JAZ motifs, is implicated to play a critical role in jasmonate signaling pathway [85]. Members of this family were reported to be strongly induced under drought conferring improved tolerance to drought and high salinity [86].

### Gene network analysis revealed tightly co-expressed modules of SRG sets

Gene coexpression networks, built using a set of microarray samples as input, can help elucidate tightly coexpressed modules that are a mixture of genes with known and unknown functions, identify hub genes, and candidate genes which can be used as biomarkers [15,87]. Using Weighted Gene Co-expression Network Analysis (WGCNA), we divided SRGs into 11, 10, 5, 8 modules of RD, RB, AD and AB, respectively, excluding a grey color module listing genes that did not significantly co-express with any other group of genes (Table S7 in File S1). The module of each SRG indicated by module color, *k*_IM_ (intramodular connectivity), a measure of how well connected or co-expressed a given gene is, with respect to other genes in its module,  MM (Module Membership), a measure of module membership correlating its gene expression profile with the Module Eigengene (ME, which is the first principal component of a given module also considered as a representative of the gene expression profile of the module) [23] and p-values are given in Table S8 in File S1. 

**Figure 3:**
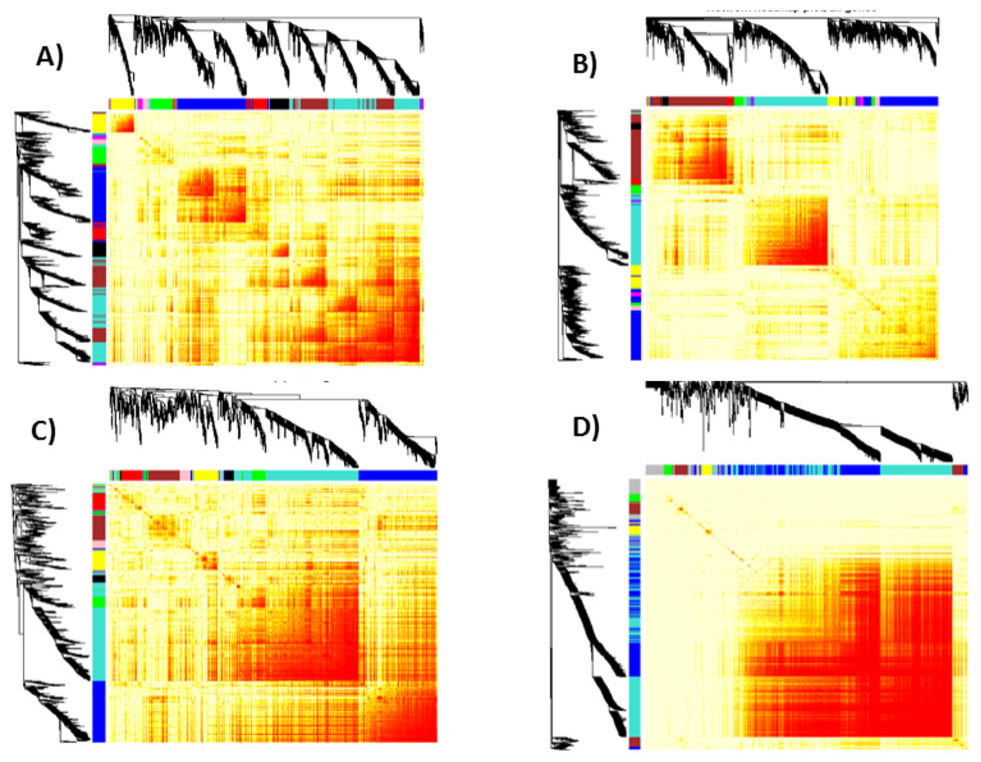
Dendrograms and heatmaps of SRGs divided into tightly co-expressed modules by the R statistical package WGCNA. A) RB, B) RD, C) AB, and D) AD. The DEGs were clustered based on co-expression patterns as represented by the dendrogram and correlation heat map. Clusters of like-regulated genes are referred to as modules and are indicated by different colors. Grey color represents the genes that could not be assigned to a module. Intensity of red coloring in the heat map indicates strength of correlation between pairs of genes on a linear scale.

**Figure 4:**
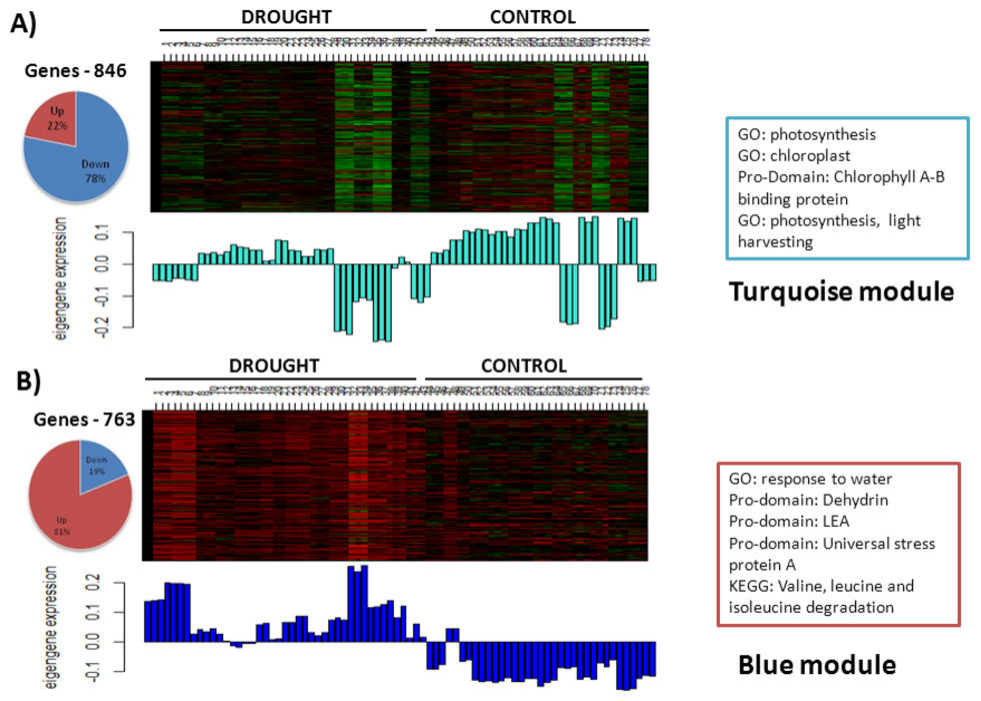
Heatmaps of turquoise and blue modules in rice under drought stress. The x-axis represents microarray samples grouped into drought treated and control samples and y-axis represents genes found in the module. Below the heatmap the corresponding module eigengene expression values are shown. The most significant functional terms found in the module are also shown. The number of genes found in each module and the percentage of up and downregulated genes in each module are shown as a pie chart.

The long length of the dendogram branches and corresponding intense red color in the heat maps of co-expression modules illustrate high co-expression of SRGs within modules and less co-expression outside the module (Figure 3). We used unsigned correlations so that positively and negatively correlated genes could be grouped into the same module. Yet, a number of modules showed high enrichment of either up or down regulated genes (Table S7 in File S1). For example, the largest module (turquoise) found in RD with 846 SRGs was made up of 663 (78%) downregulated genes while the second largest module (blue) with 763 SRGs was made up of 618 (79%) upregulated genes. We compared the 11 RD modules detected by us against 15 drought-responsive modules of rice found by another recent study using Markov Cluster (MCL) algorithm [22]. Out of those 15 modules, 14 modules were made up of 28-75% of RD SRGs, most of which significantly overlapped with one of the RD modules (Table S9 in File S1). For example, module 2 found by Zhang et al [22] was made up of 213 genes, out of which 146 (68.5%) were part of SRGs and 116 (90%) of those overlapped with RD turquoise module. The module eigengene (ME) of the RD turquoise module has low values in all drought arrays compared to control indicating that most of the genes are downregulated (green color in the heatmap) (Figure 4A). The top functional terms enriched in this module were predominantly related to photosynthesis. In the blue module, ME has higher values in all drought arrays compared to control indicating that most of the genes are upregulated (red color in the heatmap) (Figure 4B). The top functional term of blue module was ‘response to water’ followed by protein domains ‘dehydrin’ and ‘LEA’. Late embryogenesis abundant (LEA) proteins are extremely hydrophilic proteins implicated in desiccation tolerance and stabilization of proteins and membranes during drying [88]. The blue module had a very high number of TFs than turquoise (64 compared to 38 TFs) (Table S7 in File S1) although it was made up of less number of genes than turquoise. Majority of blue module TFs were from ERF and NAC families while turquoise had higher number of bZIP and CO-like TFs.

Functional enrichment analysis of each of the co-expression modules revealed a number of significant terms with FDR <0.05 (Table S10 in File S1) especially in *Arabidopsis* which were proportional to their module size. However, in rice, there was large variation in number of significant functional terms compared to module size (Table S7 in File S1). For instance, the RD module brown (size 732) had 83 significant terms but blue (size 763) had only 8 terms with FDR <0.05. Further analysis of these modules revealed higher number of genes annotated as ‘expressed protein’, ‘DUF – Domain of unknown function’ in blue module (129, 26 and 260) compared to brown module (96, 9 and 211). There were 51 and 278 genes in blue and brown modules, respectively, with high intramodular connectivity (*k*_IM_ value >100), out of which 11 and 31 genes were annotated as ‘expressed protein’ in blue and brown modules, respectively. These genes would be important candidates for further investigation as they might be playing significant role in stress response.

Under same stress, a number of modules in rice and *Arabidopsis* showed relatedness in functionality, indicating conservation of co-expression of functionally related genes across species. The module AD turquoise was related to RD brown with shared terms, response to oxidative stress (GO:0006979, AD FDR=5.18E-07, RD FDR=5.32E-07) and calcium ion binding. The module AD blue was related to RD turquoise with terms photosynthesis (GO:0015979, AD FDR=9.24E-07, RD FDR=2.0E-19) and other similar terms. AB yellow was related to RB magenta with shared terms, ‘aromatic compound biosynthetic process’ and ‘cellular amino acid biosynthetic process’. RB red with 203 upregulated genes out of 206, had 26 TFs which is double the percent of TFs found in other modules. Most of the TFs in this module belong to WRKY and MYB families with the top gene being a MYB TF, LOC_Os04g43680. The only downregulated genes in this module are LOC_Os05g37820 (major facilitator family transporter), LOC_Os09g35010 (dehydration-responsive element-binding protein) and LOC_Os02g51910 (cytokinin-O-glucosyltransferase 2). 

Among the modules found in AD, brown (size 64) had 63 upregulated genes and 22 TF genes (34%), and showed enrichment of 44 functional terms including response to various hormones and endogenous stimuli like water deprivation, salt, cold, temperature and chitin. There were 6 TFs including WRKY33 and WRKY40 in the top 10 genes in this module based on *k*_IM_ values. Among AB modules, yellow module with 90% (72 out of 80) downregulated genes and 18.75% of TFs showed enrichment of a number of terms related to secondary metabolic process including biosynthesis of aromatic compounds, flavonoids and phenylpropanoids. 

### Consensus co-expression modules of drought and bacterial stresses

**Figure 5:**
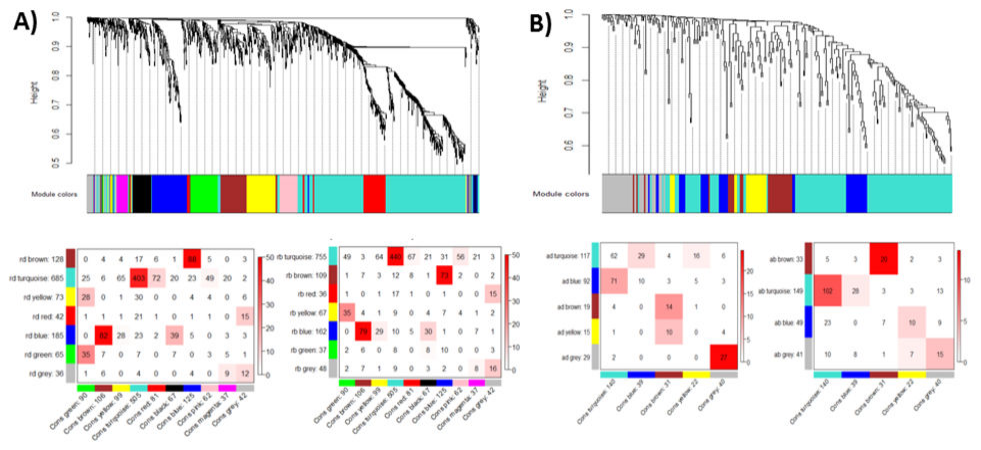
Clustering dendrogram of genes and consensus modules found in A) rice and B) Arabidopsis. The correspondence between consensus modules and modules found individually in drought and bacterial stress based on the expression values of the common genes are also shown as a table. Each row of the table corresponds to individual stress specific module (labeled by color as well as text along with the number of genes in the module), and each column corresponds to one consensus module. Numbers in the table indicate gene counts in the intersection of the corresponding modules. Coloring of the table encodes -log(p), with p being the Fisher's exact test p-value for the overlap of the two modules. The stronger red color indicates more significant overlap.

The expression profiles of the SRGs common to drought and bacterial stresses was utilized to detect consensus modules that would reveal sets of genes with similar co-expression patterns in both the stresses. We found 9 and 4 consensus modules (excluding grey module for genes that did not co-express with others) based on 1214 and 272 SRGs differentially expressed both in drought and bacterial stress in rice and *Arabidopsis*, respectively (Figure 5 and Table 3). The color coded tables below the dendrograms in Figure 5 show the correspondence between consensus modules and modules found individually in drought and bacterial stress revealing several of the modules with preserved module structure. Consensus modules brown, turquoise and blue in rice and turquoise and brown in *Arabidopsis* showed significant overlap with their counterparts indicating the module structure in drought and bacterial stress to be very similar. A complete list of SRGs with their consensus modules and *k*_ME_ values which is a measure of module membership by correlating its gene expression profile with its module eigengene is given in Table S11 in File S1.

**Table 3:** List of consensus co-expression modules found in each stress gene set.

**Rice Modules**	**Module Size**	**No. of TFs**	**RD Down/Up**	**RB Down/Up**	**Conservation of gene expression (%)**
**Brown**	106	10	8/98	4/102	94.34
**Red**	81	2	72/9	75/6	93.83
**Yellow**	99	8	32/67	39/60	90.91
**Turquoise**	505	19	396/109	363/142	82.38
**Blue**	125	5	114/11	88/37	72.80
**Green**	90	5	17/73	33/57	48.89
**Pink**	62	2	57/5	28/34	46.77
**Black**	67	7	62/5	2/65	7.46
**Magenta**	37	3	37/0	1/36	2.70
**Arabidopsis Modules**			**AD Down/Up**	**AB Down/Up**	
**Turquoise**	140	14	63/77	98/42	53.57
**Blue**	39	6	22/17	20/19	64.10
**Brown**	31	7	2/29	1/30	90.32
**Yellow**	22	0	7/15	0/22	68.18

**Figure 6:**
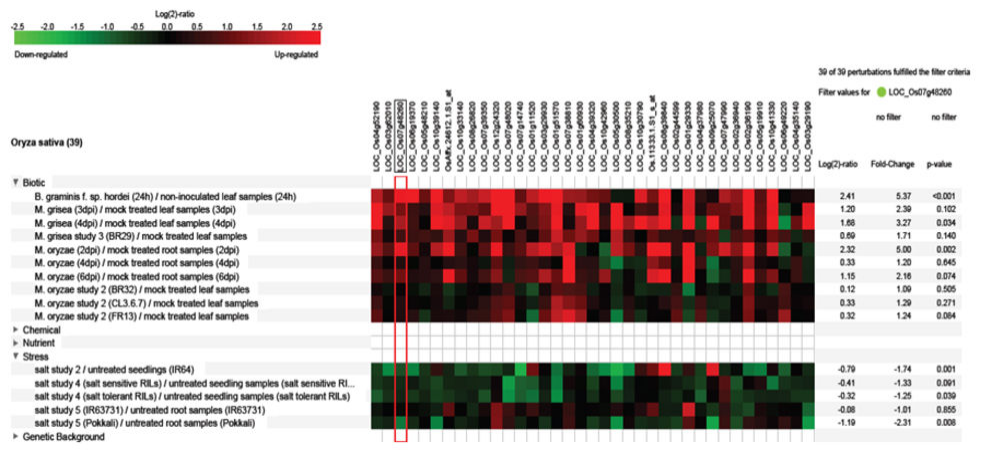
Gene expression profile of rice consensus module magenta under fungal and salt stresses using the tool Genevestigator. The heatmap shows color coded values based on Log(2)-ratio of test/control samples in different studies. A brief description of test/control samples including tissue, treatment and cultivar is also given. The top TF gene WRKY47 (LOC_Os07g48260) is highlighted with a red box and the corresponding log(2)-ratio, fold change and p-values across different microarray studies are shown.

Among the 9 consensus modules found in rice, three modules showed conservation of differential expression in >90% of genes. Of these, module red contains majorly downregulated genes while brown contains upregulated genes. Red module was enriched with terms ‘ribonucleoprotein’ and ‘rotamase’ and brown was enriched with terms ‘valine, leucine and isoleucine degradation’ and ‘NAM protein’. Interestingly, two modules (magenta and black) showed >92% of genes with reversed expression status suggesting that these set of genes possibly play a co-ordinated role specific to the stress condition. Most of the genes in these modules were downregulated under drought but upregulated under bacterial stress elucidating the differences in abiotic and biotic stress responses. We further investigated if this trend can be observed in other stresses, using the tool Genevestigator [89]. Analysis of the expression profile of genes in magenta module under salt (3 microarray studies) and fungal (3 studies and 2 pathogens, *B. graminis* and *M. oryzae*) stress conditions identified most of the genes to be highly up and downregulated under fungal and salt stresses, respectively (Figure 6). Magenta color module showed enrichment of GO terms ‘electron transport’ and ‘oxidoreductase activity’ and black was significantly enriched in the following protein domains: ‘Glycoside hydrolase, chitinase active site’, ‘DNA-binding WRKY’, ‘Bet v I allergen’ and ‘VQ’. Bet v 1 belongs to plant pathogenesis-related proteins (PR-10) family that is involved in plant development and defense systems via interactions with plant hormones [90]. VQ is a small motif found only in plants. A recent study has shown that VQ motif containing proteins act as co-activators of WRKY33 in *Arabidopsis* as part of plant defense response [91,92]. The gene LOC_Os01g61080 (WRKY24) which is an ortholog of WRKY33 of *Arabidopsis* was also part of black module. Occurrence of VQ motif containing genes (LOC_Os05g44270 and LOC_Os03g20440) and WRKY24 in the same module and upregulation of all three under bacterial stress and downregulation under drought stress suggests these genes play a similar role in rice. 

Among the *Arabidopsis* consensus modules, brown and yellow were made up of mostly upregulated genes. In brown module, 28 (90%) out of 31 SRGs were upregulated in both the stresses. It contained four WRKY TF genes including WRKY33 (AT2G38470) which was also found in rice consensus module black. The top three SRGs of brown module based on *k*_IM_ values are AT3G23250 (MYB15), AT2G22880 (VQ motif-containing protein) and AT3G25780 (allene oxide cyclase 3), which is one of the enzymes involved in jasmonic acid bioysnthesis. The top three SRGs found in yellow module are AT5G67340 (armadillo/beta-catenin repeat family protein) which functions in ubiquitin-protein ligase activity, AT4G01700 (chitinase family protein) and AT5G50200 (wound-responsive gene 3), which encodes a high-affinity nitrate transporter. 

**Figure 7:**
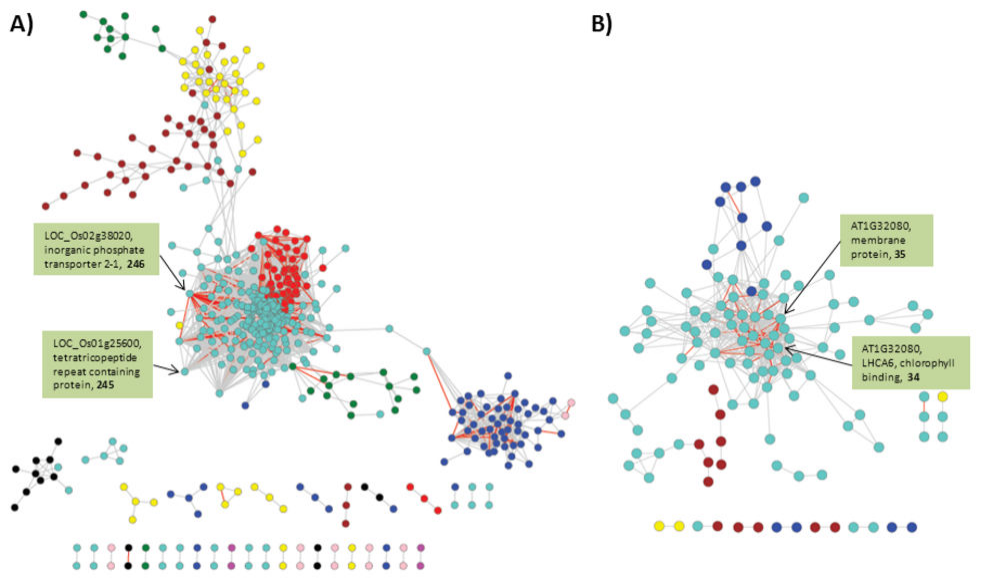
Coexpression network of SRGs common to drought and bacterial stresses. (A) rice (B) *Arabidopsis*. Nodes are color coded based on consensus modules found by WGCNA. Edges are constructed between genes with correlation coefficient (r) >0.8. The edges with r >0.8 are show in red. The gene IDs, description and number of edges of top 2 nodes in each of the networks are shown in green colored boxes.

We further analyzed the consensus co-expression modules by constructing a network based on co-expressed genes with high absolute Pearson correlation coefficient (r >0.8) in both drought and bacterial stresses. There were 16,576 edges between 585 co-expressed genes in rice (Figure 7A and Table S12A in File S1). One of the top edges was between LOC_Os02g43790, an ethylene-responsive TF and LOC_Os02g41510, a MYB TF with r >0.98 in both stresses. Color coding of nodes in network with their consensus module color showed clear grouping of genes from the same module with high number of intra-modular edges. For example, majority of blue consensus module genes had edges within the group and was largely isolated from all other modules. This indicates that these set of genes are co-regulated and exhibit stress specific co-functionality. Gene ontology analysis revealed enrichment of terms ‘cytoplasmic membrane-bounded vesicle’ (FDR=0.003) and ‘endopeptidase activity’ (FDR=0.02). Interestingly, these blue module genes were connected to the largest module (turquoise) via only one gene, LOC_Os05g09724 a HAD (haloacid dehalogenase) superfamily phosphatase which are involved in diverse housekeeping and secondary metabolism activities [93]. Red module showed the highest percent of genes (71 out of 81 or 87.6%) with a number of edges having r >0.8 in both the stresses. Black module had 15 genes (out of 67 or 22%) including 5 TFs with edges showing r >0.8, all of which showing non-conserved expression status between drought and bacterial stresses. In *Arabidopsis*, there were 509 edges between 119 genes showing r >0.8 in both stresses. Color coding the nodes with consensus module colors revealed that most of the edges were between genes of turquoise module (Figure 7B and Table S12B in File S1). The top most co-expressed genes were AT3G51420 (Strictosidine synthase-like 4) and AT1G70760 (Chlororespiratory reduction 23) with r >0.98 in both the stresses.

Out of the 642 orthologous SRGs between rice and *Arabidopsis*, 92 were part of 585 genes showing high coexpression in rice and 49 were part of 119 genes showing high coexpression in *Arabidopsis*. Out of these, 15 orthologs were part of genes showing high coexpression in both rice and *Arabidopsis* (Table 4) including four genes (PEX11B, ACA1, CYCP2 and CRR23) that showed conserved downregulation in both stresses in both the species. The genes peroxisomal biogenesis factor 11 (PEX11) and cyclin P2 had high number of co-expression edges in both the stresses. Four genes including two TFs (MYB and NAC) were upregulated in three or more stress genesets. The orthologous genes LOC_Os01G72530 and AT1G76650 coding for calcium sensor proteins showed conserved upregulation in both the stresses. AT1G76650 was previously shown to be involved in abiotic and biotic stresses such as wound and metal stress response [94,95].

**Table 4:** Orthologous SRGs with high co-expression edges and their expression status in drought and bacterial stresses.

**TAIR ID (AT)**	**TAIR annotation**	**MSU ID (LOC_Os)**	**MSU annotation**	**No. of edges (rice)**	**No. of edges (Arabidopsis**)	**AD**	**AB**	**RD**	**RB**
3G47430	PEX11B	04G45210	Peroxisomal biogenesis factor 11	220	28	Down	Down	Down	Down
4G15440	HPL1 (Hydroperoxide lyase 1); electron carrier/ heme binding / iron ion binding / monooxygenase	02G02000	Cytochrome P450	217	3	Up	Down	Down	Down
3G52720	ACA1 (Alpha carbonic anhydrase 1); carbonate dehydratase/ zinc ion binding	02G33030	Bifunctional monodehydroascorbate reductase and carbonic anhydrasenectarin-3	197	4	Down	Down	Down	Down
3G21870	CYCP2;1 (cyclin p2;1); cyclin-dependent protein kinase	04G53680	Cyclin	184	14	Down	Down	Down	Down
1G42550	PMI1 (Plastid movement impaired1)	09G38090	Expressed protein	130	15	Up	Down	Down	Down
1G19150	LHCA6; chlorophyll binding	09G26810	Chlorophyll A-B binding protein	116	34	Up	Down	Down	Down
4G16146	Protein_coding	03G19220	Expressed protein	28	2	Up	Down	Down	Down
5G22580	Protein_coding	11G05290	Stress responsive A/B Barrel domain containing protein	24	1	Down	Up	Down	Down
5G13930	TT4 (Transparent testa 4); naringenin-chalcone synthase	11G32650	Chalcone synthase	14	1	Up	Down	Down	Down
3G23250	MYB15 (MYB domain protein 15); DNA binding / transcription factor	02G41510	MYB family transcription factor	7	2	Up	Up	Down	Up
1G76650	Calcium-binding EF hand family protein	01G72530	OsCML31 - Calmodulin-related calcium sensor	6	1	Up	Up	Up	Up
3G04070	Anac047 (*Arabidopsis* NAC domain containing protein 47); transcription factor	03G21060	No apical meristem protein	4	2	Down	Up	Up	Up
5G22630	ADT5 (arogenate dehydratase 5)	04G33390	Prephenate dehydratase domain containing protein	3	2	Up	Up	Down	Up
1G70760	CRR23 (chlororespiratory reduction 23)	05G28090	Expressed protein	1	33	Down	Down	Down	Down
4G23290	Protein kinase family protein	07G35370	TKL_IRAK_DUF26-lc.15 - DUF26 kinase	1	21	Down	Down	Down	Up

## Conclusion

In this study, we performed meta-analysis of microarray studies and identified differentially expressed genes in rice and *Arabidopsis* from a wide variety of samples under drought and bacterial stresses. This type of approach enhances sensitivity in the identification of important stress response genes that could be missed by studies that are limited to specific tissue or developmental stage or level of stress. Comparative analysis of the DEGs identified common stress responsive genes between stresses and across species. Functional enrichment analysis revealed the biological processes, cellular pathways and transcription factor families that are commonly and exclusively altered under different stresses. The knowledge gained in this study on various molecular mechanisms like biosynthesis of secondary metabolites and stress specific roles of plant hormones vastly adds on to our understanding of stress response and its regulation. Weighted gene co-expression network analysis divided genes into individual and consensus modules and revealed sets of genes with conserved and reversed expression status. A number of genes with high connectivity, conserved expression but with poor annotation were identified. We propose these genes as potential candidates for stress response engineering. 

## Supporting Information

Figure S1 **Histogram plot of intensities from probe sets of rice under bacterial stress studies before and after inter-quartile range filtering and intensity filtering.**
(TIF)

Figure S2 **Analysis of network topology for various soft-thresholding powers.** Y-axis indicates scale-free fit index as a function of the soft-thresholding power (x-axis). The red line indicates soft power at which the scale-free fit index cut-off value 0.8 is reached.
(TIF)

Figure S3 **Distribution of up and downregulated genes and GO terms in different SRG sets.** A) Number of up and downregulated genes found in each stress B) Number of significant GO terms and C) Number of transcription factor (TFs) genes found in up and downregulated genes of each stress. D) Number of common SRGs showing conserved gene expression status between drought and bacterial stress. Both Down and Both Up indicate genes with conserved expression status and, D (Drought) Down - B (Bacteria) Up and D Up – B Down indicate genes with non-conserved expression pattern.
(TIF)

Figure S4 **Conservation of expression status of orthologous SRGs between rice and *Arabidopsis*.**
(TIF)

Figure S5 **Four way Venn diagram comparing significant GO terms found in A) up and B) downregulated SRG sets.**
(TIF)

Figure S6 **Significant KEGG pathways identified by the functional enrichment analysis tool DAVID in different SRG sets.** X-axis shows fold enrichment of the pathway by comparing number of genes of a pathway found in SRG set to total number of genes in pathway found in the genome. Pathways found significant (p-value <0.05) in A) upregulated SRG sets and B) downregulated SRG sets. RDU: Rice Drought Up, RBU: Rice Bacteria Up, ADU: Arabidopsis Drought Up, ABU: Arabidopsis Drought Up, RDD: Rice Drought Down, RBD: Rice Bacteria Down, ADD: Arabidopsis Drought Down, ABD: Arabidopsis Drought Down.
(TIF)

File S1 Description GEO series and samples. Table S2: List of differentially expressed genes. Table S3: Orthologous DEGs between rice and Arabidopsis. Table S4: List of significant GO terms. Table S5: List of significant KEGG orthology (K.O) terms found in various up and downregulated gene sets below hypergeometric p-value ≤0.05. Table S6: Number of TFs belonging to different TF families in in up and downregulated stress gene sets. Table S7: List of co-expression modules found in each stress gene set. Table S8: Modules of each SRG set along with their kIM (intramodular connectivity), MM (Module Membership) and p-values. Table S9: Comparision of RD modules against those detected by Zhang et.al. (2012) by MCL. Table S10: List of significant functional terms associated with genes in different co-expression modules. Table S11: List of DEGs in consensus modules along with their module membership (kME) values, z-score and associated p-values. Table S12: Common genes between drought and bacterial stresses showing correlation coefficient r >0.8.
(XLSX)
