# Supplementary figures and images for: Genes and Co-Expression Modules Common to Drought and Bacterial Stress Responses in Arabidopsis and Rice
Source: PLoS One. 2013 Oct 10;8(10):e77261. doi: 10.1371/journal.pone.0077261 (PMC3795056; doi:10.1371/journal.pone.0077261)

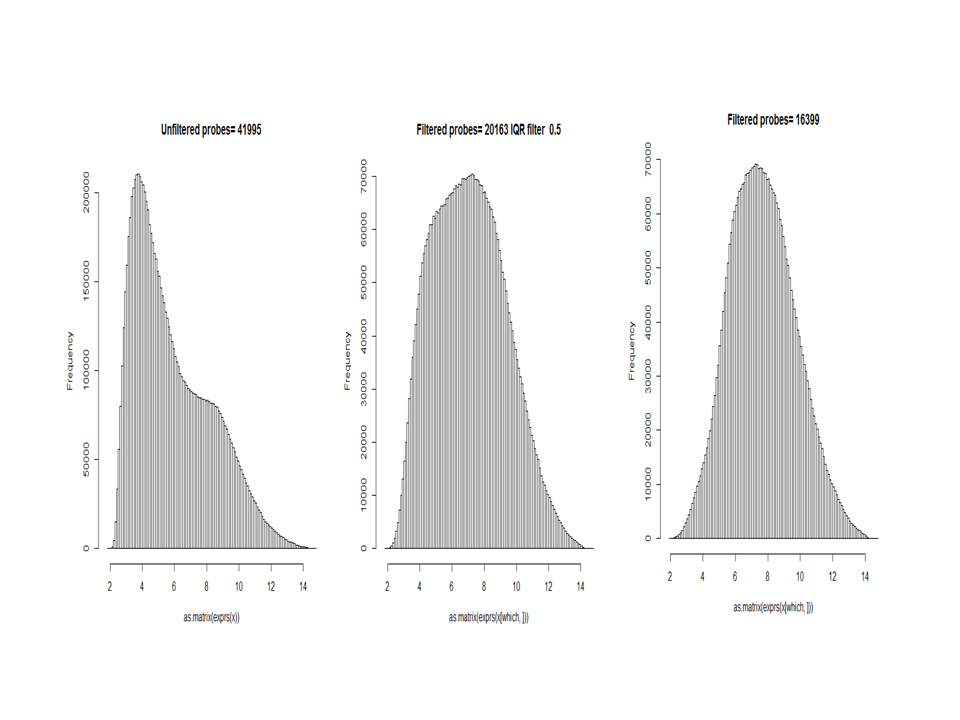

Supplement: Figure S1 — Histogram plot of intensities from probe sets of rice under bacterial stress studies before and after inter-quartile range filtering and intensity filtering. (TIF) [file pone.0077261.s001.tif]

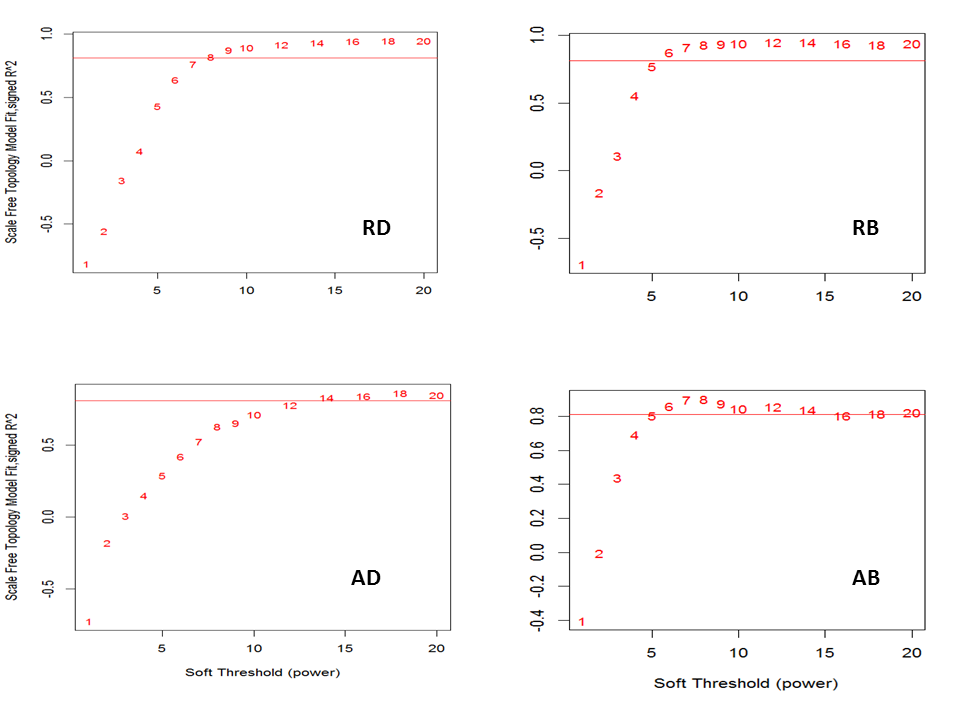

Supplement: Figure S2 — Analysis of network topology for various soft-thresholding powers. Y-axis indicates scale-free fit index as a function of the soft-thresholding power (x-axis). The red line indicates soft power at which the scale-free fit index cut-off value 0.8 is reached. (TIF) [file pone.0077261.s002.tif]

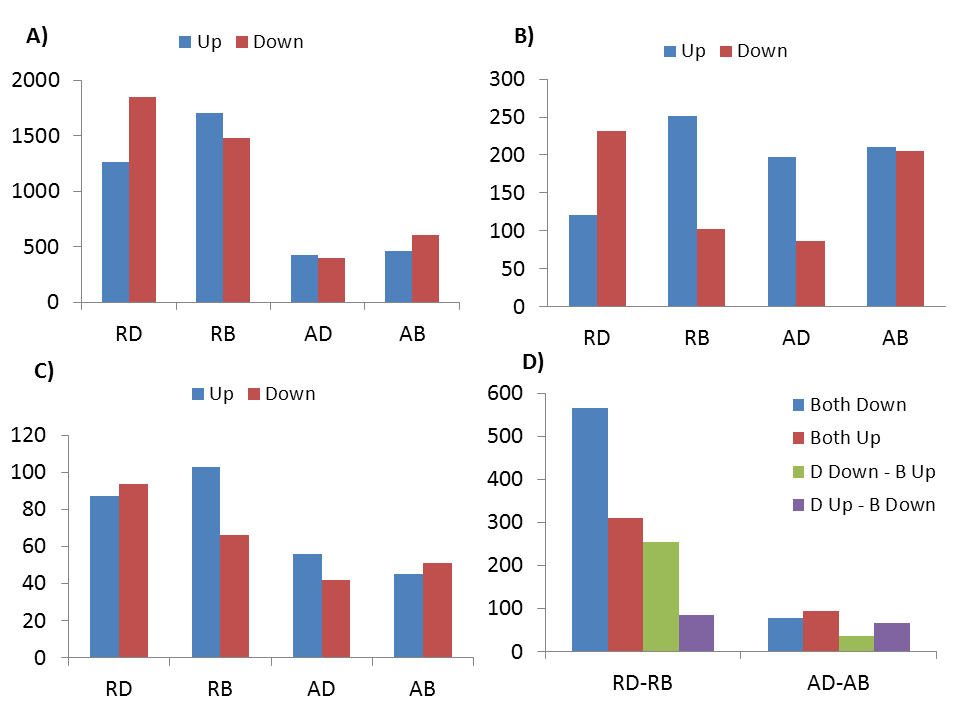

Supplement: Figure S3 — Distribution of up and downregulated genes and GO terms in different SRG sets. A) Number of up and downregulated genes found in each stress B) Number of significant GO terms and C) Number of transcription factor (TFs) genes found in up and downregulated genes of each stress. D) Number of common SRGs showing conserved gene expression status between drought and bacterial stress. Both Down and Both Up indicate genes with conserved expression status and, D (Drought) Down - B (Bacteria) Up and D Up – B Down indicate genes with non-conserved expression pattern. (TIF) [file pone.0077261.s003.tif]

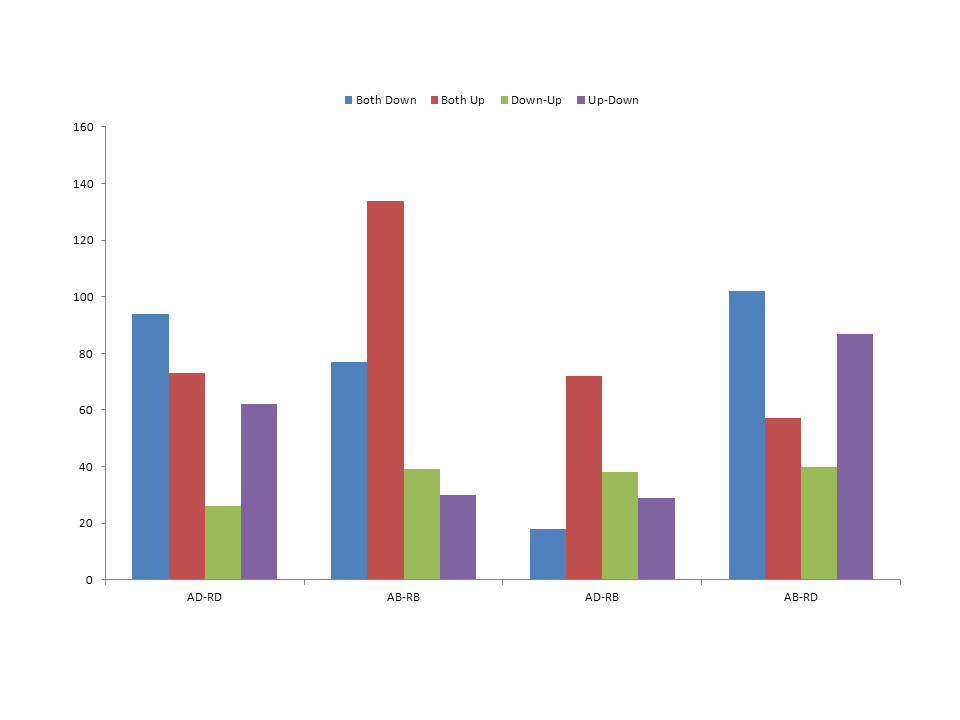

Supplement: Figure S4 — Conservation of expression status of orthologous SRGs between rice and Arabidopsis. (TIF) [file pone.0077261.s004.tif]

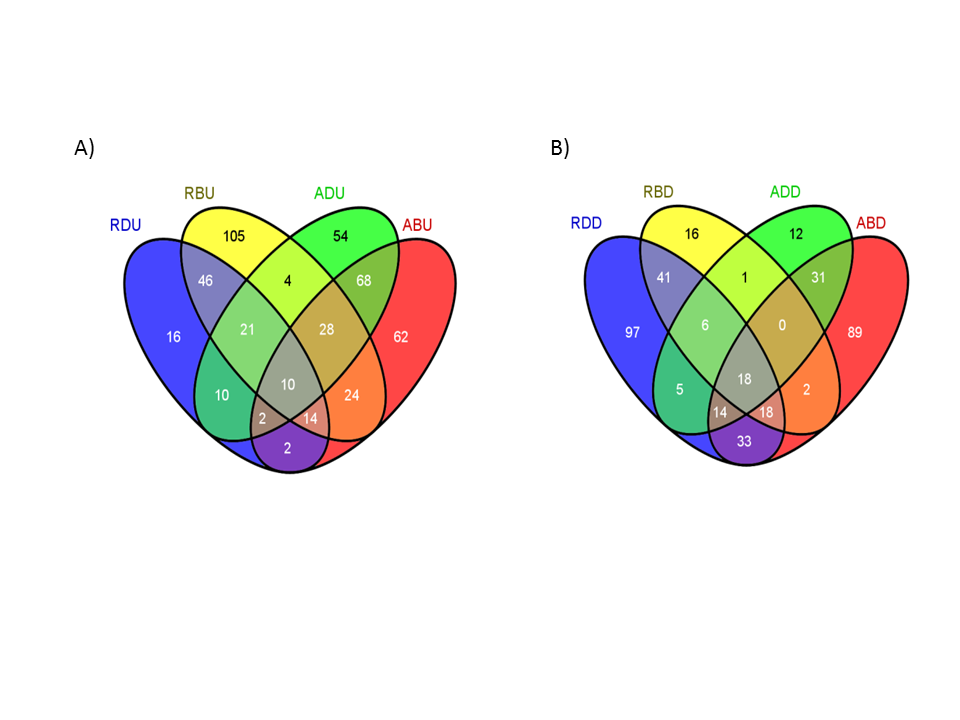

Supplement: Figure S5 — Four way Venn diagram comparing significant GO terms found in A) up and B) downregulated SRG sets. (TIF) [file pone.0077261.s005.tif]

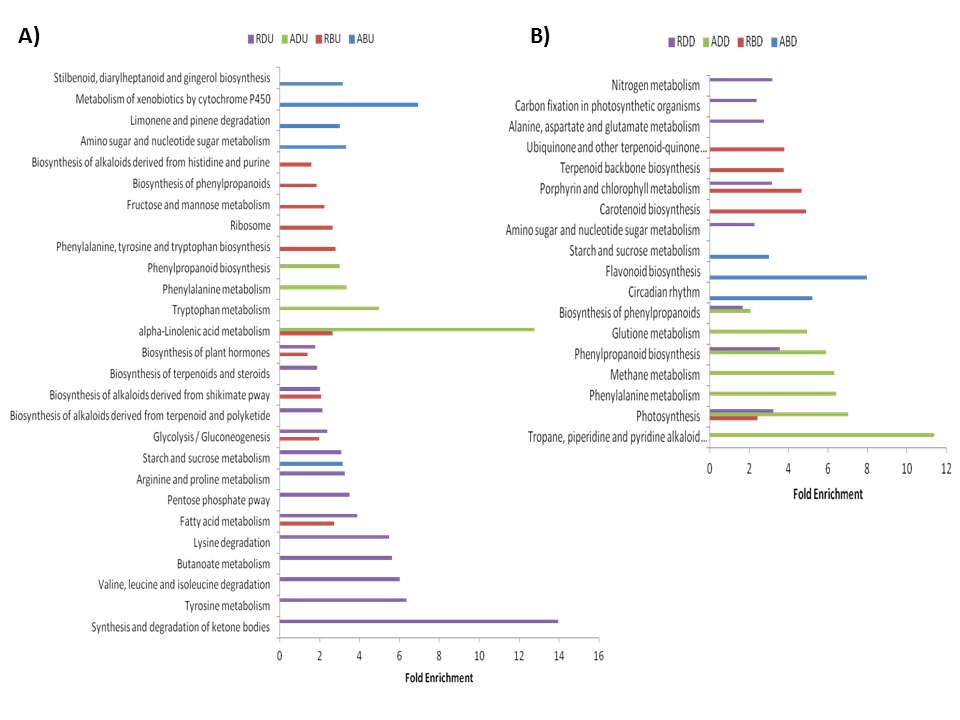

Supplement: Figure S6 — Significant KEGG pathways identified by the functional enrichment analysis tool DAVID in different SRG sets. X-axis shows fold enrichment of the pathway by comparing number of genes of a pathway found in SRG set to total number of genes in pathway found in the genome. Pathways found significant (p-value <0.05) in A) upregulated SRG sets and B) downregulated SRG sets. RDU: Rice Drought Up, RBU: Rice Bacteria Up, ADU: Arabidopsis Drought Up, ABU: Arabidopsis Drought Up, RDD: Rice Drought Down, RBD: Rice Bacteria Down, ADD: Arabidopsis Drought Down, ABD: Arabidopsis Drought Down. (TIF) [file pone.0077261.s006.tif]
